# 83. Predictors of Undetectable HDV RNA 48 weeks after completion of finite treatment with Bulevirtide and Pegylated-Interferon Alpha 2a

**DOI:** 10.1093/ofid/ofae631.020

**Published:** 2025-01-29

**Authors:** Pavel Bogomolov, Tatyana Stepanova, Viacheslav Morozov, Tarik Asselah, Fabien Zoulim, Adrian Streinu-Cercel, George Sebastian Gherlan, Olga Sagalova, Dana Tedesco, Renee-Claude Mercier, Lei Ye, Dmitry Manuilov, Audrey Lau, Marc Bourliere, Vladimir Chulanov

**Affiliations:** M.F. Vladimirsky Moscow Regional Research and Clinical Institute, Moscow, Russian Federation, Moscow, Moskva, Russia; LLC Clinic of Modern Medicine, Moscow, Russian Federation, Moscow, Moskva, Russia; LLC Medical Company Hepatolog, Samara, Russian Federation, Samara, Samara, Russia; Hopital Beaujob APHP, Universite de Paris, INSERM, Clichy, Ile-de-France, France; Hospital Croix Rousse, Lyon, Rhone-Alpes, France; Matei Bals National Institute of Infectious Diseases, Bucharest, Romania; "Carol Davila" University of Medicine and Pharmacy, Bucharest, Bucuresti, Romania; "Carol Davila" University of Medicine and Pharmacy, Bucharest, Romania; Dr. Victor Babes Foundation, Bucharest, Bucuresti, Romania; South Ural State Medical University, Chelyabinsk, Russian Federation, Chelyabinsk, Moskva, Russia; Gilead Sciences, Inc., Foster City, California; Gilead Sciences, Inc., Foster City, California; Gilead Sciences, Inc., Foster City, California; Gilead Sciences, Inc., Foster City, California; Gilead Sciences, Inc., Foster City, California; Hopital Saint Joseph, Marseille, Provence-Alpes-Cote d'Azur, France; Sechenov University, Moscow, Moskva, Russia

## Abstract

**Background:**

Chronic Hepatitis Delta (CHD) is the most severe form of viral hepatitis. Bulevirtide (BLV) is a first-in-class entry inhibitor approved in the EU for the treatment of compensated CHD. In MYR204, a Phase 2b study evaluating finite treatment with BLV with or without pegylated interferon alfa-2a (Peg-IFNa), combination treatment with 10mg BLV resulted in higher undetectable HDV RNA rates 24 weeks (W) post end of treatment (EOT) compared with either monotherapy regimen. Here, we present predictors of undetectable HDV RNA at 48W (FU-48) post-EOT.

**Table 1.**
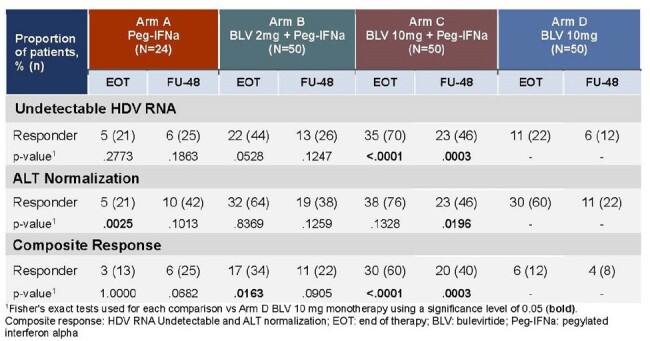
Comparison of Key Efficacy Endpoints at EOT vs FU-48

**Methods:**

174 patients with CHD were randomized (1:2:2:2) with stratification of cirrhosis status and received (A) Peg-IFNa for 48W; or (B) BLV 2mg + Peg-IFNa, or (C) BLV 10mg + Peg-IFNa for 48W followed by 48W of monotherapy with BLV 2mg or 10mg, respectively; or (D) BLV 10mg for 96W. All patients were followed up to FU-48. Efficacy endpoints were compared by Fisher’s exact test. The logistic regression model examined if any baseline (BL) characteristics predicted responses at FU-48 with arms B and C. Characteristics with p< 0.05 were considered potential predictors. The MMRM was used to evaluate treatment effect on HDV RNA during initial 48W on-treatment.

**Figure 1.**
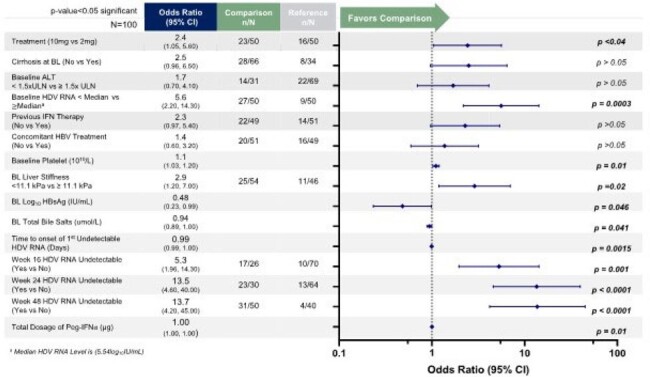
Selected Characteristics as Potential Predictors of undetectable HDV RNA at FU-48 in patients treated with BLV +Peg-IFNa.

Logistic regression analysis was performed on a merged data set of patients that were treated with combination therapy of BLV (either 2 or 10mg) +Peg-IFNa to evaluate predictors of HDV RNA undetectability. P values <0.05 are represented in bold.

**Results:**

BL characteristics were similar between all arms. Key endpoints such as, HDV RNA undetectability, ALT normalization, and composite response (HDV RNA undetectable and ALT normalization) at EOT and FU-48 are shown in Table 1. Selected potential BL predictors of undetectable HDV RNA at FU-48 included BL HDV RNA < median of 5.54 log_10_ IU/mL (Odds Ratio (OR): 5.6, p=0.0003), and BL liver stiffness < 11.1kPa (OR: 2.9, p< 0.02) (Fig.1). Time to onset of HDV RNA undetectability (OR: 0.99, p=0.0015), and undetectable by on-treatment 24W (OR:13.5, p< 0.0001) predicted HDV RNA undetectability at FU-48.

BLV (2 or 10mg) + Peg-IFNa had a higher weekly decline of HDV RNA compared to Peg-IFNa (LS-means difference: -0.0414 log_10_IU/mL or -0.0542 log_10_IU/mL (p< 0.0001), respectively) within the first 48W on-treatment.

**Conclusion:**

In patients with CHD treated with a finite regimen of BLV + Peg-IFNa early rapid on-treatment viral decline and achievement of HDV RNA undetectability are associated with undetectable HDV RNA at FU-48.

**Disclosures:**

**Tarik Asselah, PhD**, Abbvie: Expert Testimony|Abbvie: Investigator|Eiger Pharmaceuticals: Expert Testimony|Eiger Pharmaceuticals: Investigator|Gilead Sciences, Inc.: Expert Testimony|Gilead Sciences, Inc.: Investigator|Janssen: Expert Testimony|Janssen: Investigator|Merck: Expert Testimony|Merck: Investigator|Myr pharmaceutical: Expert Testimony|Myr pharmaceutical: Investigator|Roche: Expert Testimony|Roche: Investigator **Fabien Zoulim, MD, PhD**, Aligos Therapeutics: consulting fees|Antios Therapeutics: consulting fees|Assembly Biosciences: Grant/Research Support|Assembly Biosciences: consulting fees|Beam Therapeutics: Grant/Research Support|Gilead Sciences, Inc.: consulting fees|Janssen: Grant/Research Support **Dana Tedesco, PhD**, Gilead Sciences, Inc.: Employee|Gilead Sciences, Inc.: Stocks/Bonds (Private Company) **Renee-Claude Mercier, PharmD**, Gilead Sciences, Inc.: Employee|Gilead Sciences, Inc.: Stocks/Bonds (Private Company) **Dmitry Manuilov, MD**, Gilead Sciences, Inc.: Employee|Gilead Sciences, Inc.: Stocks/Bonds (Private Company) **Audrey Lau, MD, PhD**, Gilead Sciences, Inc.: Employee|Gilead Sciences, Inc.: Stocks/Bonds (Private Company) **Marc Bourliere, MD, PhD**, AbbVie: Board Member|AbbVie: Expert Testimony|Gilead Sciences, Inc.: Board Member|Gilead Sciences, Inc.: Expert Testimony|Intercept: Board Member|Intercept: Expert Testimony|Roche: Board Member|Roche: Expert Testimony **Vladimir Chulanov, MD, PhD**, AbbVie: Advisor/Consultant|AbbVie: Expert Testimony|AstraZeneca: Advisor/Consultant|AstraZeneca: Expert Testimony|Bristol Myers Squibb: Advisor/Consultant|Bristol Myers Squibb: Expert Testimony|Gilead Sciences, Inc.: Advisor/Consultant|Gilead Sciences, Inc.: Expert Testimony|GSK: Advisor/Consultant|GSK: Expert Testimony|Hepatera: Advisor/Consultant|Hepatera: Expert Testimony|Merck Sharp & Dohme: Advisor/Consultant|Merck Sharp & Dohme: Expert Testimony|Roche: Advisor/Consultant|Roche: Expert Testimony|R-Pharm: Advisor/Consultant|R-Pharm: Expert Testimony

